# Transcriptomic Responses of *Rhizobium phaseoli* to Root Exudates Reflect Its Capacity to Colonize Maize and Common Bean in an Intercropping System

**DOI:** 10.3389/fmicb.2021.740818

**Published:** 2021-10-28

**Authors:** José Luis Aguirre-Noyola, Mónica Rosenblueth, Michel Geovanni Santiago-Martínez, Esperanza Martínez-Romero

**Affiliations:** ^1^Programa de Ecología Genómica, Centro de Ciencias Genómicas, Universidad Nacional Autónoma de México, Cuernavaca, Mexico; ^2^Department of Biochemistry and Molecular Biology, The Pennsylvania State University, University Park, PA, United States

**Keywords:** RNA-seq, milpa system, agriculture, polygalacturonase, proline, carbonic anhydrase, symbiotic nitrogen fixation

## Abstract

Corn and common bean have been cultivated together in Mesoamerica for thousands of years in an intercropping system called “milpa,” where the roots are intermingled, favoring the exchange of their microbiota, including symbionts such as rhizobia. In this work, we studied the genomic expression of *Rhizobium phaseoli* Ch24-10 (by RNA-seq) after a 2-h treatment in the presence of root exudates of maize and bean grown in monoculture and milpa system under hydroponic conditions. In bean exudates, rhizobial genes for nodulation and degradation of aromatic compounds were induced; while in maize, a response of genes for degradation of mucilage and ferulic acid was observed, as well as those for the transport of sugars, dicarboxylic acids and iron. Ch24-10 transcriptomes in milpa resembled those of beans because they both showed high expression of nodulation genes; some genes that were expressed in corn exudates were also induced by the intercropping system, especially those for the degradation of ferulic acid and pectin. Beans grown in milpa system formed nitrogen-fixing nodules similar to monocultured beans; therefore, the presence of maize did not interfere with *Rhizobium*–bean symbiosis. Genes for the metabolism of sugars and amino acids, flavonoid and phytoalexin tolerance, and a T3SS were expressed in both monocultures and milpa system, which reveals the adaptive capacity of rhizobia to colonize both legumes and cereals. Transcriptional fusions of the *putA* gene, which participates in proline metabolism, and of a gene encoding a polygalacturonase were used to validate their participation in plant–microbe interactions. We determined the enzymatic activity of carbonic anhydrase whose gene was also overexpressed in response to root exudates.

## Introduction

Associated maize (*Zea mays*) and common bean (*Phaseolus vulgaris*) crops have been extensively used in agriculture for thousands of years in Mesoamerica. These associated crops are called “milpa” (from the Nahuatl words: “milli” which means cultivated plot and “pan” which means upon) (Rodríguez-Robayo et al., 2020). Squash plants, as well as other plant species, are frequently included; however, they are not in such a close association as that existing with maize and bean (Lopez-Ridaura et al., 2021). Bean usually climbs on maize and climbing beans have long cycles similar to maize cycles and have been identified as having a high nitrogen-fixing capacity in comparison to bush *P. vulgaris* plants, which are used in monoculture. Milpa systems have a high agricultural value for farmers as they provide a large food volume per area and a diet rich in nutrients, vitamins and bioactive compounds (Méndez-Flores et al., 2021; Novotny et al., 2021). In polyculture, maize has a higher yield per plant than in monoculture and each bean plant has the same or higher yield depending on the variety planted (Santalla et al., 2001; Zhang et al., 2014). Additional studies have determined that in milpa, the use of soil is more efficient than in monocultures (Aguilar-Jiménez et al., 2019). A reason for this is that maize has a deeper root system than beans, which allows the milpa to explore a greater volume of soil to absorb nutrients and water (Albino-Garduño et al., 2015). Maize–bean intercropping is also more effective in controlling diseases and insect pests (Fininsa, 1996) and soil fertility is conserved better than in monoculture crops (Alfonso et al., 1997).

Since the Green Revolution around 1940–1970, with the advent of fertilizers, increase of irrigation, herbicides, hybrid varieties, and mechanization, maize and bean started to be cultivated as monocultures in many places, especially in large-scale farms (Aguilar-Jiménez et al., 2019). The use of land in monoculture has brought the degradation of soils and the dependence on external inputs. If more land were cultivated in milpa system, the biodiversity, richness of the soils, and the use of diverse landraces that are suitable for milpa could be recovered.

The microbiota of soils where milpa is cultivated in different areas of Mexico has been studied, with Proteobacteria, Actinobacteria, and Verrucomicrobia as the most abundant phyla (Rebollar et al., 2017; Aguirre-von-Wobeser et al., 2018; Gastélum and Rocha, 2020). By growing together, with roots intermingled, maize and bean could share microbiota in milpa. We surmised this possibility and searched for rhizobia in maize plants associated with bean. We found rhizobia as natural maize endophytes inside stems and roots (Gutiérrez-Zamora and Martínez-Romero, 2001). Among plant-associated bacteria, rhizobia deserve a special position due to their capacity to induce the formation of legume root nodules where rhizobia perform nitrogen fixation once differentiated to bacteroids. A population analysis from maize rhizobia revealed that *R. phaseoli* strain Ch24-10 (hereby called Ch24-10) belonged to a group of bacteria abundantly found in maize plants (Rosenblueth and Martínez-Romero, 2004), thus we choose Ch24-10 for further studies. In beans, Ch24-10 exhibits high nitrogen-fixing capabilities, as does *R. phaseoli* strain CIAT 652. However, CIAT 652, which is used for commercial inoculant production, lacks a plasmid that encodes carbon-substrate usage genes, so Ch24-10 could be best for plant colonization.

Nitrogen fixation in legumes contributes to a substantial input of nitrogen in agriculture but also in natural habitats and it is desirable to extend nitrogen fixation to cereals (Rosenblueth et al., 2018). Though maize does not form nodules, maize exudates stimulated rhizobial nodulation and nitrogen fixation of faba beans (*Vicia faba*) by enhancing the expression of legume root genes involved in flavonoids synthesis, auxin signaling, and the nodulation process (Li et al., 2016). Better nodulation performance and acquisition of nutrients were also observed in maize associated with bean (Cardoso et al., 2007), soybean (*Glycine max*; Nyoki and Ndakidemi, 2018), and pea (*Pisum sativum*; Zhao et al., 2020).

Root exudates play an important role in early stages of plant colonization by providing metabolites that act as nutrients, chemoattractants, or antimicrobials for soil microbes (Lopez-Guerrero et al., 2013). Transcriptome analysis of rhizobacteria exposed to root exudates for a short time has made it possible to detect early adaptive responses that would be masked in prolonged experiments due to the microbial metabolism or reabsorption of root metabolites (Phillips et al., 2004; Shidore et al., 2012; Xie et al., 2015; Yi et al., 2018). In fact, the bacterial genes involved in the metabolism of some carbohydrates and amino acids are downregulated in maize root exudates after 48 h (Zhang et al., 2015).

We wondered if there was an advantage for *Rhizobium* in an associated crop and we supposed that in this condition, there would be a richer composition of exudates that would be available to rhizobia. An RNA-seq analysis from *R. phaseoli* Ch24-10 stimulated by exudates of bean and maize grown in monoculture or associated in milpa could help to address this issue. Previous transcriptomic studies analyzed Ch24-10 in maize roots after a few days or in the presence of other bacteria (Gómez-Godínez et al., 2019). We performed here a short exposure (2 h) to exudates that has the advantage that nutrients would not be depleted as in long-term assays and a fast response to the different plant exudates could be detected.

## Materials and Methods

### Bacterial Strain and Preparation of Inoculum

*Rhizobium phaseoli* strain Ch24-10 was isolated from corn stems of a milpa system in Cholula, Puebla, Mexico. In maize, it colonizes the rhizosphere and roots as an endophyte (Rosenblueth and Martínez-Romero, 2004) and has plant growth-promoting activities (Matus-Acuña et al., 2018). In symbiosis with bean, Ch24-10 forms nitrogen-fixing nodules. Ch24-10 was cultured in PY broth (5 g peptone, 3 g yeast extract, and 0.6 g CaCl_2_ per liter) for 24 h (exponential phase) at 30°C with continuous shaking. Cultures were centrifuged for 5 min at 4,025 × *g* and cell pellets were washed once with 10 mM MgSO_4_ for use in the plant experiments.

### Plants and Experimental Design

**FIGURE 1:**
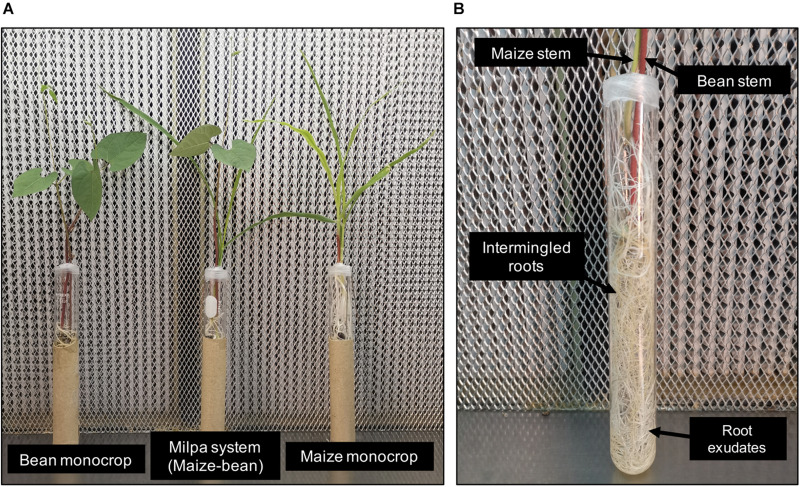
Hydroponic system to obtain root exudates. **(A)** Maize and bean growing in monoculture and milpa. **(B)** Bean and maize roots in the milpa assay.

Common bean (variety Negro Jamapa) and maize (landrace “Negro criollo” from Hidalgo, Mexico) seeds were surface disinfected with 70% ethanol (1 min), 1.2% sodium hypochlorite (20 min), washed five times with distilled water), and 2% sodium thiosulfate (2 min and rinsed two times with distilled water) as described in Rosenblueth and Martínez-Romero (2004). Disinfected seeds were germinated on agar–water plates and subsequently placed on stainless steel pedestals into glass tubes (25 × 200 mm) containing 50 mL of N-free Fahraeus solution [0.132 g CaCl_2_, 0.12 g MgSO_4_⋅7H_2_O, 0.1 g KH_2_PO_4_, 0.075 g Na_2_HPO_4_⋅2H_2_O, 0.005 g Fe-citrate, and 0.07 mg each of MnCl_2_⋅4H_2_O, CuSO_4_⋅5H_2_O, ZnCl_2_, H_3_BO_3_, and Na_2_MoO_4_⋅2H_2_O per liter of milli-Q (Type 1 Ultrapure water)]. Each tube contained two seedlings: bean–bean, maize–maize, and bean–maize (milpa system) and they were maintained for 5 days at 28°C with a 16/8-h light/dark photoperiod in axenic conditions. The lower part of the hydroponic system was covered with kraft paper which allows roots to grow in darkness and the aerial part in the presence of light (Figure 1) as recommended by Silva-Navas et al. (2015). Plant roots with 30 mL of exudates were inoculated at a final concentration of 10^8^ Ch24-10 cells mL^–1^. After 2 h of root–bacteria interaction, 10% (*v*/*v*) of RNA later reagent (Ambion) was added to all tubes before RNA extraction. Each experimental replicate included root exudates from three tubes. Three replicates were considered for all treatments and for the controls without plant (N-free Fahraeus solution). In addition, 1 mL from each root exudate was plated in LB and PY medium and incubated for 3 days to detect microbial contamination.

### RNA Extraction and High Throughput Sequencing

Bacteria from root exudates were concentrated by centrifugation for 5 min at 4,025 × *g* at 4°C and pellets were immediately frozen in liquid nitrogen. Total RNA was extracted using RNeasy Mini Kits (Qiagen) with proteinase K (Qiagen), lysozyme (Sigma-Aldrich), and DNAse I (Sigma-Aldrich). RNA was quantified using a Nanodrop 2000 spectrophotometer (Thermo Scientific) and the RNA integrity number (RIN) of the samples was determined using a TapeStation 2200 electrophoresis system (Agilent Technologies). Our criterion for selecting suitable samples for RNA-seq was an rRNA ratio >0.8 and RIN >8. Ribosomal RNAs were removed using the Ribo-Zero Bacteria Protocol (Illumina) and cDNA libraries were prepared using the TruSeq non-stranded protocol (Illumina). The high-throughput sequencing was performed with the Illumina HiSeq 4000 platform (100 nt paired-end reads) by Macrogen Inc. (Seoul, South Korea).

### Bioinformatic Analysis

The quality of raw reads was carried out with FASTQC v0.11.8 (Andrews, 2010) and low-quality sequences and Illumina adapters were removed using Trimmomatic v0.39 (Bolger et al., 2014) with the parameters LEADING:3 TRAILING:3 SLIDINGWINDOW:4:15 MINLEN:36. Bowtie2 v2.3.5 (Langmead and Salzberg, 2012) was used to map reads against the *R. phaseoli* Ch24-10 genome (GCF_000268285.2_RPHCH2410v2) with the parameters – very-sensitive -q -p 20 -x -2 -S. The paired-end mapped reads associated with each gene were quantified using featureCounts v1.6.4 (Liao et al., 2014). The Bioconductor package edgeR v3.6.0 was used for the differential expression analysis of transcriptome data (Robinson et al., 2010). The counts per million (CPM) values were normalized using the trimmed mean of M-values method (TMM) to estimate gene expression levels. A gene was considered as differentially upregulated if it had a Log2 fold change >1 at false discovery rate (FDR) threshold of 0.95 with adjusted *p*-values ≤ 0.05 (Bustamante-Brito et al., 2019). Venn diagrams were performed to show exclusive and shared genes between treatments using the web-tool http://bioinformatics.psb.ugent.be/webtools/Venn/. The upregulated genes were classified into COG (clusters of orthologous groups) categories with the online version of eggNOG-mapper v2^1^ (Huerta-Cepas et al., 2019).

### Generation of Ch24-10 Mutants

Two Ch24-10-derivative strains (CCG-9A11 and CCG-VP1) with reporter genes were generated in this study to test the transcription of rhizobial genes that were expressed in the presence of root exudates.

For CCG-9A11, a transposon mutagenesis was performed with pCAM140 (Tn5-*gusA*; Wilson et al., 1995). Mutants that were induced with maize exudates using X-gluc and 4-MUG were selected (see below for the protocols). It was verified that there was only one Tn5 insertion by Southern hybridization using a fragment of Tn5 as a probe. To locate the site of insertion, a clone was obtained and sequenced. The insertion was in gene RPHASCH2410_PD02690 that encodes a GH28 polygalacturonase (poly-alpha-1,4- galacturonide glycanohydrolase).

For the CCG-VP1 mutant, site-directed mutagenesis of *putA* gene was performed. For this, a fragment of *putA* gene from Ch24-10 was amplified by PCR with the primers put767F (5′TTC AGTCGACGCGGCATCTATGACGGTCCTG3′) and put1527R (5′TTCAGGATCCATCAGCGCCTCGACCGAAACA3′) and cloned in pCR4-TOPO vector, following the instructions of the manufacturer (Invitrogen). After checking by gel electrophoresis that the clones had the correct size, they were sequenced to confirm that they had the fragment from *putA.* One of them was selected for transferring the fragment to the suicide vector pJQ200mp18 (Quandt and Hynes, 1993). The *lacZ* cassette (pKOK6; Kokotek and Wolfgang, 1989) was inserted in *Bgl*II site of *putA*. This construction was conjugated with Ch24-10 in a triparental cross. To select double recombinants, the transconjugants were plated in 12% of sucrose. The plasmid profile was visualized by the Eckhardt (1978) technique, modified by Hynes and McGregor (1990). All the clones were carried out in *Escherichia coli* strain DH5-α competent cells and selected with the appropriate antibiotics.

### β-Glucuronidase Assays

Corn and bean plants were grown together or in monoculture (as mentioned above) and root exudates were centrifuged for 15 min at 4,025 × *g* to remove plant debris. One milliliter of root exudates was inoculated with 10^8^ cells mL^–1^ of the Ch24-10-derivative strain CCG-9A11, which has the β-glucuronidase gene (*gusA*) as a reporter and incubated at 30°C for 24 h. According to Jefferson (1987) with modifications of Xi et al. (1999), the β-glucuronidase activity induced by root exudates was measured using a fluorometric assay with 4-MUG (methylumbelliferyl-β-D-glucuronide hydrate; Sigma-Aldrich) in a Synergy H1 Multi-Mode Microplate Reader (BioTek Instruments) at an emission/excitation wavelength of 365/460 nm. Using a methylumbelliferone (MU) reference curve, enzyme activities were reported as nmMU/min/10^6^ cells.

To detect β-glucuronidase activity within the roots, maize and bean seedlings inoculated with CCG-9A11 or Ch24-10 were cultivated for 15 days in flasks with semi-solid N-free Fahraeus (Martínez et al., 1987). The whole roots were washed twice with MQ water and placed in 50 mL tubes with X-gluc solution (5-Bromo-6-chloro-3-indolyl β-D-glucuronide cyclohexylammonium salt; Gold biotechnology) for 48 h at 28°C on a rotary shaker (Shamseldin, 2007). The blue signal inside the roots and nodules indicated gene induction, in contrast to colorless roots inoculated with Ch24-10 that does not carry the reporter gene *gusA* (Supplementary Figure 1). The biological nitrogen fixation of bean nodules in monoculture and in milpa was measured by acetylene reduction assays using gas chromatography and reported as nmol C_2_H_4_/h/plant (Martínez et al., 1985).

### β-Galactosidase Assays

For the β-galactosidase assays, CCG-VP1 was grown in minimal medium (MM) containing (per liter) K_2_HPO_4_ 3.8 g, KH_2_PO_4_ 3 g, sucrose 1 g, NH_4_Cl 1 g, MgSO_4_⋅7H_2_O 0.1 g, CaCl_2_ 0.1 g, H_3_BO_3_ 2.86 mg, ferric citrate 5 mg, MnSO_4_⋅4H_2_O 2.03 mg, ZnSO_4_⋅7H_2_O 0.22 mg, CuSO_4_⋅5H_2_O 0.08 mg, and Na_2_MoO_4_⋅H_2_O 0.08 mg at 30°C for 48 h with continuous shaking (Montes-Grajales et al., 2019). One milliliter of bacterial culture was mixed with 1 mL of maize, bean, or milpa exudates and incubated for 2 h. Later, β-galactosidase activities were measured according to Li et al. (2012) using ONPG (o-nitrophenyl-β-D-galactopyranoside; Sigma-Aldrich) and reported in Miller units (Mu). MM with proline (20 mM) was used as a positive control.

### Carbonic Anhydrase Activity Assay

Ch24-10 was incubated in 10 mL of maize, bean, and milpa exudates at 30°C and the cells were harvested at 2 and 24 h by centrifugation at 9,500 × *g* for 10 min. Cell pellets were resuspended in 50 mM Tris–HCl pH 7.5 and broken by sonication (three cycles: 15 s *plus* 60 s resting) under anaerobic conditions. Then, the cell homogenate was centrifuged at 6,080 × *g* at 4°C for 3 min and the supernatant (protein extract) was kept on ice and used immediately for carbonic anhydrase activity determination (Santiago-Martínez et al., 2016). One to two milligrams of protein extract with 1 mL anaerobic reaction buffer (45 mM Na-bicarbonate pH 6.8 plus 0.02 mM ZnCl_2_) was incubated in sealed 2 mL bottles with a rubber stopper at 25°C. Buffer was previously bubbled with nitrogen for 30 min, and the air in the headspace of each bottle was replaced by nitrogen to maintain the anaerobic conditions. To detect the CO_2_ formation, 5 μL of the headspace was taken and injected at different times (0, 30, 60, and 120 s) in a GC-2010-Shimadzu gas chromatograph equipped with a capillary column HP-PLOT/U of 30 m length, 0.32 mm I.D. and 10 μm film (Agilent, United States), and a thermal conductivity detector. Commercial α-carbonic anhydrase from bovine erythrocytes (Sigma-Aldrich) was used as a positive control (modified from Veitch and Blankenship, 1963; Lira-Silva et al., 2012). Values of CO_2_ formed in assay reaction buffer with no enzyme and with boiled enzyme were subtracted from values of CO_2_ formed by Ch24-10 samples. Carbonic anhydrase activity was shown as nmol CO_2_ produced/min/mg protein.

### Statistical Analysis

Student’s *t*-test was used to compare the fresh weight and nitrogenase activity of bean nodules from monoculture and milpa. One-way ANOVA and a Tukey’s honestly significant difference test were performed for multiple comparison analysis. The package agricolae v1.3.5 in R studio was used for statistical analysis considering a *p*-value ≤ 0.05 as statistically different (de Mendiburu and de Mendiburu, 2019).

## Results

**FIGURE 2:**
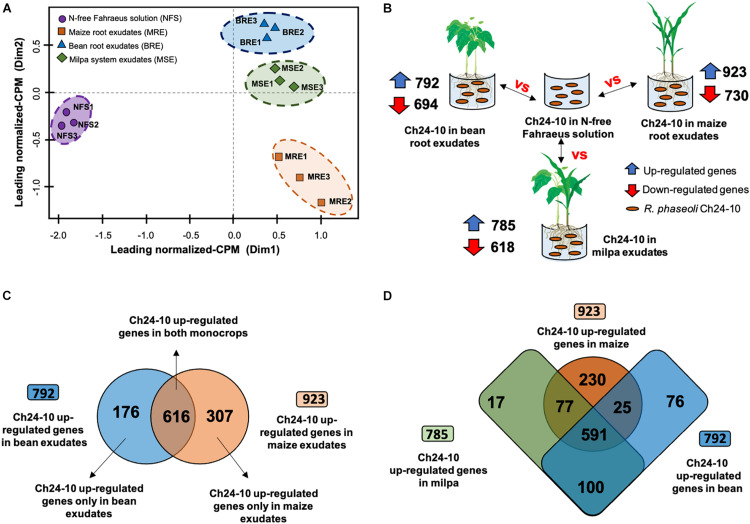
Differential expression analysis of transcriptomic data of *R. phaseoli* Ch24-10. **(A)** Multidimensional scale (MDS) analysis of RNA-seq transcriptome profiles of Ch24-10 in root exudates and Fahraeus solution. Counts per million (CPM) were normalized using the trimmed mean of M-values (TMM) method. **(B)** Differentially expressed genes of Ch24-10 in root exudates compared to Fahraeus solution. A gene was considered as differentially expressed at false discovery rate (FDR) threshold of 0.95, adjusted *p*-values ≤ 0.05, and Log2 fold change >1 (upregulated) or <1 (downregulated) using the package edgeR v3.6.0. **(C)** Venn diagram between upregulated genes of Ch24-10 induced by bean and maize exudates. **(D)** Venn diagram between upregulated genes of Ch24-10 expressed in maize and bean monocultures, and milpa. Venn diagrams were generated using the web-tool available at http://bioinformatics.psb.ugent.be/webtools/Venn/ to show exclusive and shared genes between treatments.

To identify rapid rhizobial responses in a 2-h exudate exposure to bean and maize root exudates from monoculture or milpa system (Figure 1), a whole transcriptome analysis of *R. phaseoli* Ch24-10 was performed under four conditions: with bean root exudates, maize roots exudates, root exudates from milpa, and N-free Fahraeus solution. Because bacterial populations inhabiting the rhizoplane have different metabolisms depending on the area of the root that they colonize (Kragelund et al., 1997), transcriptomes of bacteria growing in plant exudates offer the advantage of being more homogeneous and reproducible. In our study, an average of 75 million paired and mapped reads was recovered from each triplicate of the four treatments. More than 98% of RNA-seq reads was assigned to Ch24-10 genome. The multidimensional scale (MDS) analysis of Figure 2A shows that the replicas are grouped close to each other into four groups that correspond to the treatments. The transcriptomic data in Fahraeus solution differed greatly from those obtained in plant exudates; in turn, the transcriptomic profiles from bean exudates and milpa exudates are close to each other, but distant from those of maize root exudates.

**FIGURE 3:**
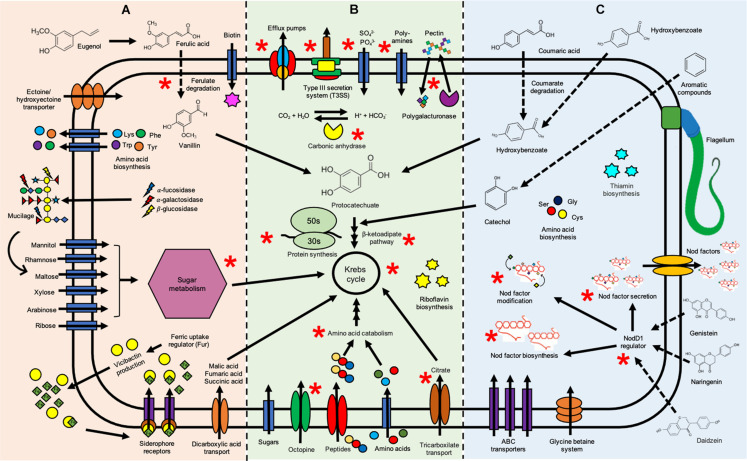
Schematic representation of functions and metabolic pathways of *Rhizobium phaseoli* induced by root exudates. The orange zone **(A)** highlights the rhizobial functions associated with corn exudates, the blue zone **(C)** shows functions in the presence of bean exudates, and the green zone **(B)** indicates shared functions in both conditions. Ch24-10 metabolic processes induced by milpa exudates are denoted by a red asterisk. The information used here was obtained from transcriptome data in the presence of beans (Supplementary Table 1), maize (Supplementary Table 2), and milpa (Supplementary Table 3) and from data about chemical composition of exudates reported in the literature (Bolaños-Vásquez and Werner, 1997; Carvalhais et al., 2011; Fan et al., 2012; Tawaraya et al., 2014; Zhang et al., 2020).

Differentially overexpressed genes were found in *R. phaseoli* incubated 2 h in root exudates (see Figure 2B) from common bean (Supplementary Table 1), from maize (Supplementary Table 2), and from milpa (Supplementary Table 3). *R. phaseoli* transcripts of Fahraeus solution were used to distinguish genes that were expressed as a consequence of hydroponic components and not by the presence of plant roots; the differentially expressed genes under this condition will not be reviewed here. A metabolic model in response to exudates was proposed to show the main metabolic pathways and cellular functions of Ch24-10 in the rhizosphere of corn, bean, and milpa system (Figure 3). The Ch24-10 genes that participate in these adaptative processes are detailed below for each of the exudate treatments.

### Rhizobial Genes Expressed Only in Bean Root Exudates

**FIGURE 4:**
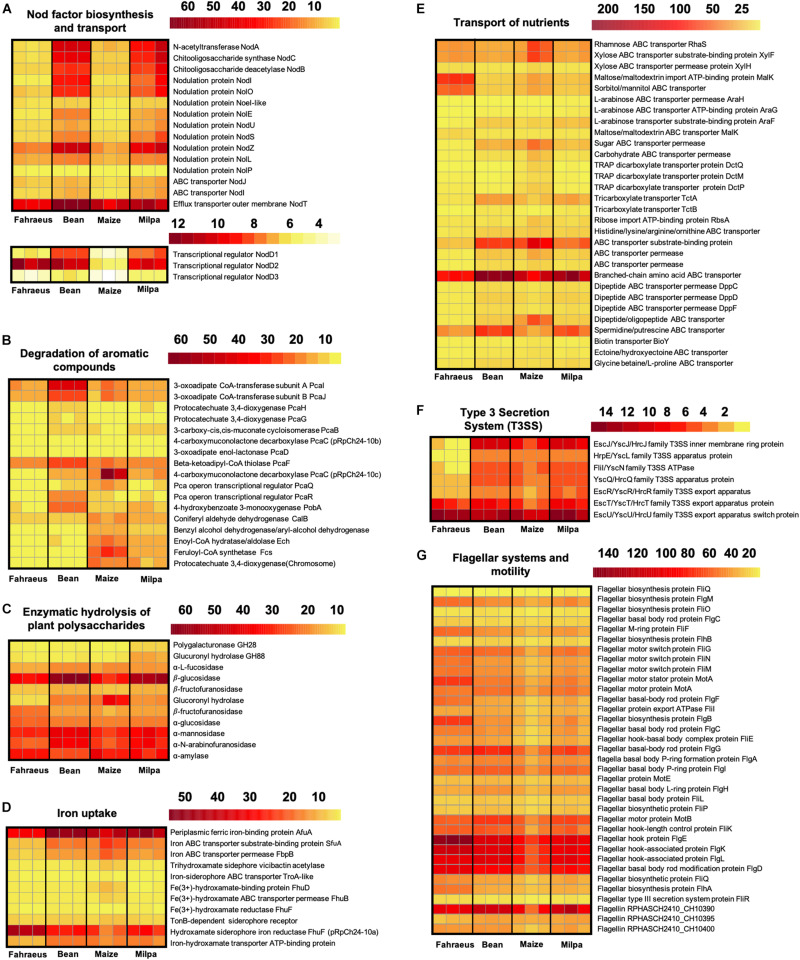
Expression of rhizobial genes induced by root exudates. Heat maps showing expression levels of genes involved in **(A)** Nod factor biosynthesis and transport, **(B)** degradation of aromatic compounds, **(C)** enzymatic hydrolysis of plant carbohydrate polymers, **(D)** iron uptake, **(E)** transport of nutrients, **(F)** Type 3 secretion system (T3SS), and **(G)** flagellar systems and motility. Color scales indicate Log2CPM that were obtained by the trimmed mean of M-values normalization method using the package edgeR v3.6.0. The transcriptional profiles in triplicate for each treatment are shown.

In *P. vulgaris* exudates, 176 Ch24-10 genes were highly expressed in comparison to maize exudates (Figure 2C). They were distributed in the chromosome (66%), chromid pRpCh24-10d (17%), symbiotic plasmid (pSym) pRpCh24-10c (7%), plasmid pRpCh24-10b (5%), and plasmid pRpCh24-10a (5%). Among them, we detected *nodA*, *nodB*, and *nodC* genes necessary for Nod factor synthesis. *nodS*, *nodU*, *nodZ*, *nolO*, *noeI*, and *nolL* genes encoding enzymes that modify Nod factors through methylation, carbamoylation, fucosylation, or acetylation were also upregulated, as well as the genes *nodI*, *nodJ*, and *nodT* involved in the secretion of Nod factors (Figure 4A). Consistent with these findings, three regulatory *nodD* genes were found in Ch24-10, *nodD1* being the most highly expressed.

*pobA* gene encodes a p-hydroxybenzoate hydrolase involved in the formation of protocatechuate from hydroxybenzoate, which is one of the main plant cell wall-bound phenolics (Sircar and Mitra, 2008). Genes from the *pca* regulon and the positive regulator *pcaR* for protocatechuate catabolism were induced by beans (Figure 4B). PcaR also has a role in chemotaxis towards aromatic compounds (Romero-Steiner et al., 1994). 4-oxalocrotonate tauromerase that oxidizes benzene, toluene, and xylene was differentially expressed. We also found an increase in ABC transporter genes that were reported to be induced by bean exudates in *Rhizobium tropici* (Rosenblueth et al., 1998) and by osmotic stress, like the transport system for glycine betaine and proline similar to ProVWX from *E. coli* (Lucht and Bremer, 1994).

### Rhizobial Genes Expressed Only in Maize Root Exudates

Three hundred seven genes were upregulated in maize exudates in comparison to bean exudates (Figure 2C), 209 overexpressed genes were found in the chromosome, 48 genes in the chromid, 23 genes in pRpCh24-10b, 21 genes in pRpCh24-10a, and only 6 genes in pSym pRpCh24-10c. Genes involved in the transport of arabinose, rhamnose, xylose, maltose, mannitol, and biotin (Figure 4E) and for mucilage degradation like α-fucosidase, α-galactosidase, and β-glucosidase (Figure 4C) genes were found overexpressed. Ch24-10 with maize exudates also showed activation of the DctPQM transporter for C4-dicarboxylates and its two-component regulatory system DctB/DctD.

An increased expression of genes to degrade eugenol (*calA* and *calB*) and ferulic acid (*fcs* and *ech*; Figure 4B) and for the synthesis of the siderophore vicibactin and its transport were observed as well (Figure 4D). Three copies of the *dapA* gene encoding the dihydrodipicolinate synthase of the lysine biosynthesis pathway were detected as upregulated; the same case for genes encoding chorismate and anthranilate synthases that could contribute to phenylalanine, tyrosine, and tryptophan production. Moreover, maize exudates also induced the overexpression of genes for Lrp/AsnC family transcriptional regulator commonly activated by exogenous amino acids and for the ferric uptake regulatory protein (Fur) which controls iron homeostasis and siderophore biosynthesis (Hassan and Troxell, 2013).

### Rhizobial Genes Induced by Bean or Maize Monocultures

When Ch24-10 transcripts from bean exudates were compared to those from maize exudates, 616 upregulated genes were found in both conditions (Figure 2C and Supplementary Table 4). Most of these genes were located on the chromosome (72%) and the chromid (13%) and a few of them in plasmids pRpCh24-10b (7%), pRpCh24-10a (4%), and the pSym pRpCh24-10c (4%).

Among the genes with the highest expression, we identified those encoding ABC transporters for sugars, amino acids, and polyamines, and for nitrate, sulfate, and phosphates as well as for *tctABC* and *occQMPT* genes necessary to import tricarboxylates and octopine, respectively (Figure 4E). Another finding was the high level of expression of the *dppBCDF* operon that is responsible for the import of di/tripeptides. Oligopeptide transporter genes have been found expressed after Ch24-10 is maintained for a few days in both bean and maize roots (López-Guerrero et al., 2012). Genes for key enzymes involved in the catabolism of lysine, histidine, glutamate, and threonine were also highly expressed, for example, genes *hutH*, *hutU*, *hutI*, *hutF*, and *hutG* that comprise entirely the two main routes for histidine utilization.

Gene induction was observed for several efflux pumps such as RmrAB, AcrAB-TolC, EmrAB-TolC, and MATE (Multidrug and Toxic Compound Extrusion) and for a Type 3 secretion system (T3SS) that resembles the Ysc-Yop virulence apparatus of *Yersinia enterocolitica* (Figure 4F). Genes for the assembly of the flagellar motor, hook and basal complex, and for the biosynthesis of flagellin were found expressed in all transcriptomes. However, more flagellar transcripts were recovered with *P. vulgaris* exudates than in maize exudates (Figure 4G).

In both root exudates, many genes encoding ribosomal proteins were upregulated including S1, S2, from S3 to S15, and from S18 to S21 associated with the 30S subunit and from L1 to L6, L9, L10, L11, L13, L14, from L18 to L25, L27, L28, and from L31 to L36 associated with the 50S subunit, as well as genes encoding aminoacyl-tRNA ligases for methionine, cysteine, aspartate, tyrosine, phenylalanine, histidine, and isoleucine. We also detected transcriptional regulator genes with high expression such as those belonging to the FadR, DeoR/GlpR, TetR/AcrR, and ROK families. These transcriptional factors orchestrate physiological responses associated with sugar metabolism, quorum sensing, multidrug resistance, and extrusion of toxic compounds, among many others.

### Transcriptomic Profiles of *R. phaseoli* in Milpa

Seven hundred eighty-five Ch24-10 genes were found highly expressed in milpa in comparison to transcriptomic data in Fahraeus solution (Figure 2B and Supplementary Table 3). Among them, 566 upregulated genes were found in the chromosome, 104 in the chromid, 43 in pRpCh24-10b, and 36 in both pRpCh24-10a and pSym pRpCh24-10c.

**FIGURE 5:**
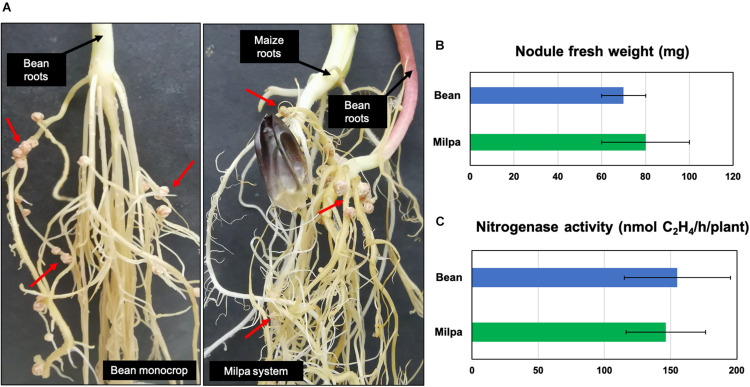
Nodulation in bean monocrop and milpa. **(A)** Nitrogen-fixing nodules of bean plants 15 days after inoculation with *R. phaseoli* Ch24-10. Nodules are shown by red arrows. Comparison of **(B)** fresh weight and **(C)** nitrogenase activity of bean nodules from monoculture and milpa.

Another interesting comparison was Ch24-10 transcripts in milpa against those from bean exudates and maize exudates, where we found 42 and 38 differentially expressed genes, respectively (Supplementary Tables 5, 6). The transcriptomic profiles observed in milpa resemble those from bean exudates, partly due to the nodulation genes (synthesis, modification, and transport of Nod factors), which presented similar levels of expression in both conditions (Figure 4A). Bean in milpa formed nitrogen-fixing nodules after 15 days of inoculation with Ch24-10 in semi-solid medium (Figure 5A) and we found no statistically significant differences comparing against the fresh weight and nitrogen fixation of nodules from bean in monoculture (Figures 5B,C). Unexpectedly maize exudates significantly induced *nodA* gene expression (Log2 fold change = 1.4) but not at levels that were observed with both plants together in the milpa (Log2 fold change = 4.5) and bean exudates (Log2 fold change = 4.7; Supplementary Table 4). Genes for the degradation of protocatechuate, ferulic acid (Figure 4B), and plant carbohydrate polymers (Figure 4C) expressed in milpa were similarly expressed in maize exudates.

On the other hand, 591 expressed genes in milpa were found in transcriptomes from monocrops (Figure 2D and Supplementary Table 4). For example, genes encoding ABC transporters for sugars, amino acids, octopines, and iron were detected as well as genes encoding enzymes for the production of ornithine, putrescine, and homospermidine. Likewise, the expression of genes for the catabolism of amino acids such as histidine, tyrosine, and phenylalanine was observed. Furthermore, many of the upregulated genes in all exudates were associated with COG functional categories such as transport and metabolism of carbohydrates, amino acids and inorganic ions, transcription, translation, and ribosome biogenesis (Supplementary Figure 2). An increased level of expression of Ch24-10 genes encoding proteins for the extrusion of toxic plant metabolites (RmrAB, EmrAB, and AcrAB systems) was observed in root exudates either from monocultures or milpa, as well as genes encoding the SecYEG translocon, the SecDF protein-export membrane protein and components from a T3SS.

**FIGURE 6:**
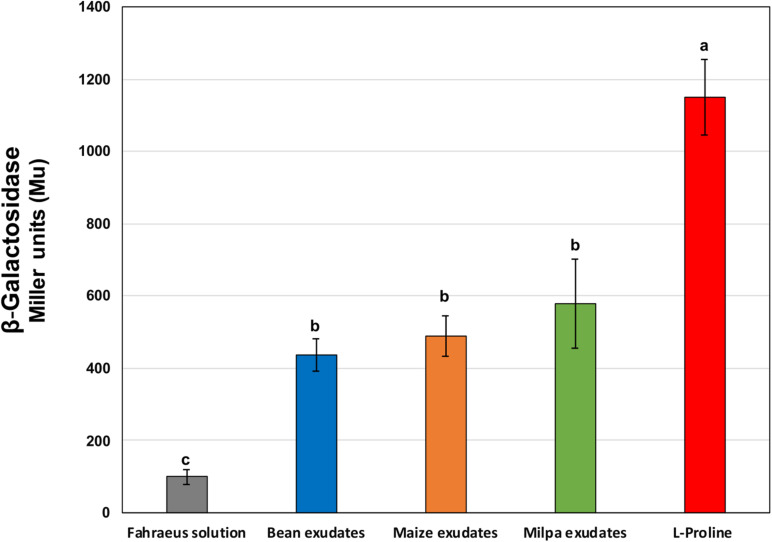
β-galactosidase activities of CCG-VP1 in response to root exudates for 2 h. CCG-VP1 is Ch24-10 containing the *lacZ* reporter gene adjacent to *putA* gene. β-galactosidase activity was determined with a colorimetric assay using ONPG. Different lowercase letters between treatments show statistically significant differences (*p*-value ≤ 0.05) according to ANOVA followed by a Tukey’s honestly significant difference test. Three replicates for each treatment were performed.

Rhizobial genes encoding several transporters for proline were highly expressed in all root exudates. We generated strain CCG-VP1 that is a mutant of Ch24-10 affected in the *putA* gene encoding a bifunctional proline dehydrogenase/pyrroline-5-carboxylate dehydrogenase involved in proline catabolism (Liu et al., 2017), thus, CCG-VP1 is not able to grow in MM with L-proline as sole carbon and nitrogen source (Supplementary Figure 3). The expression of *putA* was evaluated using the β-galactosidase activity of CCG-VP1; *putA* gene was induced in the presence of exudates from bean, maize, and milpa and also when proline was added to the MM as a positive control (Figure 6).

**FIGURE 7:**
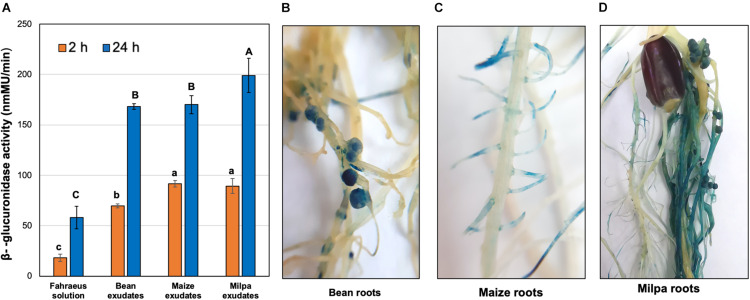
β-glucuronidase activity of CCG-9A11 in response to plants. CCG-9A11 is Ch24-10 with a *gusA* gene fusion upstream of the polygalacturonase gene. **(A)** β-glucuronidase activity induced by root exudates was measured using a fluorometric assay with 4-MUG. Three replicates for each treatment were performed. Different letters between treatments show statistically significant differences (*p*-value ≤ 0.05) according to ANOVA followed by a Tukey’s honestly significant difference test. Lowercase and uppercase letters were used to show the differences at 2 and 24 h of exposure to root exudates, respectively. β-glucuronidase activity within **(B)** bean nodules and **(C)** maize roots in monoculture and **(D)** in milpa system. The blue signal inside the roots is indicative of polygalacturonase gene induction.

Among the genes that were overexpressed in milpa compared to monocultures, we found genes encoding glucoside hydrolases for the degradation of pectin. Expression of glycoside hydrolase genes was further explored in the CCG-9A11 strain with a transcriptional *gusA* fusion in a Ch24-10 gene that encodes a GH28 polygalacturonase (poly-alpha-1,4-galacturonide glycanohydrolase). During the first 2 h of exposure to root exudates, the expression of polygalacturonase gene (reported as β-glucuronidase activity) was higher in experiments with exudates from maize and milpa than from bean (Figure 7A). However, at 24 h, β-glucuronidase activities were higher in milpa exudates (199.1 ± 16 nmMU) compared to bean (168.2 ± 3nmMU) and maize exudates (170.1 ± 9 nmMU). We inoculated CCG-9A11 onto plants and β-glucuronidase activity was detected within the bean nodules (Figures 7B,D) as well as in lateral roots of maize (Figures 7C,D), whether these were grown separately or in milpa.

RPHASCH2410_CH12295 gene encoding a chromosomal β-carbonic anhydrase was expressed under monoculture and milpa conditions and so this enzymatic activity in Ch24-10 was evaluated (Table 1 and Supplementary Figure 4). The results showed that in the milpa exudates, the carbonic anhydrase activity was higher at 2 h (794 ± 56 nmol CO_2_ produced/min/mg) compared to maize (640 ± 42 nmol CO_2_ produced/min/mg protein) and bean exudates (673 ± 45 nmol CO_2_ produced/min/mg protein), and this trend was maintained at 24 h.

**TABLE 1:** Ch24-10 carbonic anhydrase activity after treatment with root exudates for 2 and 24 h.

	Anhydrase activity (nmol CO_2_ produced/min/mg protein)			
Treatments	2 h exposure		24 h exposure	
Fahraeus solution	504 ± 26	c	534 ± 35	C
Bean exudates	673 ± 45	b	808 ± 39	B
Maize exudates	640 ± 42	b	788 ± 25	B
Milpa exudates	794 ± 56	a	1,015 ± 44	A

*Different letters between treatments show statistically significant differences, at a *p-*value ≤ 0.05, according to ANOVA and a Tukey’s honestly significant difference test. Lowercase and uppercase letters were used to show the differences at 2 and 24 h of exposure to root exudates, respectively. Numbers are the mean of three repetitions ± standard deviation.*

## Discussion

Cereal–legume associations are commonly used in agriculture with maize as a preferred cereal in combination with *P. vulgaris*, faba bean or soybean. When a cereal is combined with a legume, disease and insect pests are better controlled (Fininsa, 1996), and the soil is conserved better than in monoculture. In addition, the microbial diversity of the rhizosphere of mixed cultures is greater because root exudates act as nutrients and chemoattractants that allow microorganisms to colonize new hosts, which leads to the microbiota being shared between plants, including endophytes as rhizobia (Gutiérrez-Zamora and Martínez-Romero, 2001; Mommer et al., 2016). The ancestral maize association, milpa, originated in Mesoamerica as maize and *P. vulgaris* are native to this area. We designed a mini-milpa in the laboratory in hydroponic conditions to assess the early effects of milpa exudates on *R. phaseoli* Ch24-10, which is an outstanding *Rhizobium* strain.

Exudate composition is not a universal characteristic of a plant species, it may differ among individuals from a single species, vary with plant age, environmental factors, and nutrients available to the plant (Carvalhais et al., 2011). Metabolite exudation by maize and bean changes over time; however, some root metabolites are released at both early and late stages of plant growth (da Silva Lima et al., 2014; Tawaraya et al., 2014). Each plant could contain distinct endophytes that may transform or even produce exuded molecules. Two plant species together could exert novel selective pressures on endophytes. Thus, the outcome of exudates could be different in associated roots. In turn, changes in exudation profiles affect the metabolism of the rhizosphere microbiota (Chaparro et al., 2013; Vacheron et al., 2013). Rhizobia in roots use sugars and amino acids from exudates and would compete with pathogens besides producing plant hormones as do other plant growth-promoting bacteria (Rosenblueth and Martínez-Romero, 2006).

Maize exudes more photosynthate than bean plants, which may be due to their large C4 photosynthetic capability (Brown et al., 2005). Maize roots mainly secrete glucose, melibiose, maltose, and fructose (Fan et al., 2012). Their exudates are rich in amino acids such as aspartic acid, glutamic acid, glutamine, serine, and alanine, but are low in lysine (Carvalhais et al., 2011). Approximately 25% of the organic compounds that flow into the root system of maize is released by exudates (Haller and Stolp, 1985). A more general response of Ch24-10 to sugar and mucilage and pectin degradation may occur in maize exudate with fucosidase, galactosidase, and polygalacturonase genes highly expressed. A reporter-gene approach confirmed that polygalacturonase is highly expressed in maize exudates from monoculture or in association with bean (Figure 7A). Sugars such as galactose, fucose, mannose, arabinose, xylose, and glucose together with uronic acids like galacturonic acid and glucuronic acid are the main components of mucilage (Nazari et al., 2020). Genes for transporters and enzymes for metabolism of these sugars were expressed in maize exudates, so Ch24-10 perhaps consumes them as carbon sources once they are released from plant polymers or directly from the exudates. *Rhizobium leguminosarum* produces carbohydrases associated with its ability to grow in mucilage as the sole carbon source (Knee et al., 2001).

Several bacteria commonly use sugars and amino acids from plant exudates as the main nutrients, thereby organic acids could accumulate in the rhizosphere (Koo et al., 2005). Malic acid, fumaric acid, succinic acid, and others are secreted by maize roots (Fan et al., 2012) causing acidification of the rhizosphere as shown in Supplementary Figure 5. Maize and milpa exudates are more acidic than bean exudates from 7-day-old plants (Supplementary Figure 6), as has been described in maize–faba bean mixed crops where the rhizosphere is acidified as a consequence of an increase in the secretion of carboxylic acids and protons (Li et al., 2007). Our transcriptome data show that bacterial specialized mechanisms for sensing and transporting dicarboxylic acids were activated by maize exudates. C4-dicarboxylates are used preferentially by rhizobia and their transport is essential for effective symbiosis (Iyer et al., 2016). Organic acids can also dissociate Fe-minerals and improve iron uptake by plants, preventing chlorosis (Abadía et al., 2002). On maize roots in soil, there may exist a diverse microbial population with a consequent competition for iron. Vicibactin is a cyclic trihydroxamate siderophore originally reported in *R. leguminosarum*, which is a close relative of *R. phaseoli* (Wright et al., 2013). Here, we found that vicibactin acetylase gene was highly expressed in maize exudates suggesting that vicibactin is produced and that iron may be limiting in maize exudates.

Carbonic anhydrase enzymatic activities were quantified in cell extracts of *R. phaseoli* Ch24-10 exposed to milpa, maize, or bean exudates for 2 or 24 h. Similar activities were recorded in rhizobia from maize or bean exudates and a higher activity from rhizobial cells that were milpa exudates (Table 1). We showed that enzymatic activities reflected transcriptomic results. Carbonic anhydrase produces carbonate that rhizobia could excrete into roots and diminish exudate acidity and indirectly facilitate the cotransport of monocarboxylates with proton; however, this may not be the case because rhizobial inoculation on exudates did not modify the pH of the exudates (not shown). Therefore, carbonate may be a carbon substrate for rhizobia allowing it to profit from root derived CO_2_, and this may provide an ecological advantage for rhizobia. The role of the bacterial carbonic anhydrase in plant–microorganism interactions is still unknown; however, wheat endophytes that produce this enzyme increase plant photosynthesis and growth (Aslam et al., 2018).

We infer that ferulic acid may be an important component for rhizobial nutrition in maize exudates. Ferulic acid is an abundant phenolic acid attached to plant walls and its abundance in maize is at least ten times more than in other cereals (Bento-Silva et al., 2018). Ferulic acid is released by corn roots (Zhang et al., 2020). In bean, coumaric acid and 4-hydroxybenzoate may be preferentially used by Ch24-10 according to the level expression of *pobA* gene and *pca* operon. Coumaric acid and 4-hydroxybenzoate are abundant in bean exudates in early plant stages (Tawaraya et al., 2014). We also detected differential *R*. *phaseoli* expression of genes for chemotaxis to aromatic compounds in bean. The ability of Ch24-10 to degrade aromatic compounds from plants could provide better fitness in the rhizosphere of both crops by providing carbon sources as well as detoxification mechanisms. Other *P. vulgaris*-nodulating rhizobial strains such as *R. tropici* CIAT899 have the capacity to metabolize polycyclic aromatic hydrocarbons (González-Paredes et al., 2013).

Some amino acids like asparagine, phenylalanine, tryptophan, threonine, and valine are produced by corn and bean roots as are the organic acids citrate and fumarate (Pellet et al., 1995; Carvalhais et al., 2011; Fan et al., 2012; Tawaraya et al., 2014). We detected the expression of Ch24-10 genes encoding transporters such as those for citrate, amino acids, and peptides in bean and maize transcriptome data. Putrescine and spermidine transporters were also overexpressed in both plants. Polyamines have been found in bean exudates (Tawaraya et al., 2014), but their presence in corn exudates has not been well studied. Proline is an abundant amino acid in maize exudates (Phillips et al., 2004) and is also produced by bean roots at different growth steps (Tawaraya et al., 2014). The *putA* gene has been used as a biosensor to monitor the exudation of proline by pea roots (Rubia et al., 2020) and its overexpression in *Sinorhizobium meliloti* strains increases their competitiveness to colonize and nodule alfalfa (*Medicago sativa* L.; van Dillewijn et al., 2001). In this work, *putA* expression was induced in an exudate exposure of 2 h, but no statistically significant differences were observed between plant treatments (Figure 6). However, CCG-VP1 is not affected in maize root colonization (rhizospheric and endophytic) but is less competitive in occupying bean nodules compared to the wild strain (Supplementary Figure 7). The transcriptional regulator YjgM (OatM) genes were upregulated in maize, bean, and milpa exudates. In *Salmonella*, YigM upregulates the cysteine regulon (VanDrisse and Escalante-Semerena, 2018) and it would be interesting to further determine if Ch24-10 produces and excretes cysteine in plant roots. An exo-metabolome analysis revealed that Ch24-10 can excrete amino acids such as aspartic acid, alanine, tyrosine, and phenylalanine when it grows in MM (Montes-Grajales et al., 2019).

In the maize–faba bean intercropping system, legume roots stimulate the expression of Bx genes necessary for the biosynthesis of the phytoalexins DIMBOA (2,4-dihydroxy-7-methoxy-1,4-benzoxazin-3-one) and MBOA (6-methoxy-2-benzoxazolinone) in maize (Yan et al., 2014). Microbial diversity from the rhizosphere of maize deficient in DIMBOA or MBOA differs from that of the wild type, therefore, maize metabolites may also limit the growth of soil microbes (Cotton et al., 2019; Cadot et al., 2021). Phytoalexins produced by milpa system has not been determined; however, we detected overexpression of Ch24-10 genes *rmrA* and *rmrB* that has been described in *Rhizobium etli* as being important in the tolerance to the phytoalexin phaseolin and flavonoids of beans (González-Pasayo and Martínez-Romero, 2000). *R. phaseoli* Ch24-10 is able to grow in the presence of the MBOA phytoalexin from maize (Rosenblueth and Martínez-Romero, 2004). Genes encoding components of a T3SS were induced in Ch24-10 by monocultures and milpa system. *Rhizobium* T3SS effectors are injected in plant-host cells and have roles in symbiosis, perhaps inhibiting plant defense responses (Marie et al., 2001). T3SS structural components that encode syringe components are quite conserved while effectors may be more variable and their effects have not been completely elucidated (Nelson and Sadowsky, 2015).

Similar to our results, high levels of expression of rhizobial *nod* genes have been observed in response to *P. vulgaris* exudates which contain daidzein, naringenin, and genistein, even higher than those induced by synthetic flavonoids (Bolaños-Vásquez and Werner, 1997). Maize may synthesize several flavonoids and some of them are secreted by roots (Li et al., 2016; Jin et al., 2017). We detected that Ch24-10 *nodA* gene was upregulated in maize exudates compared to Fahraeus solution, but not as strongly as in bean exudates. A *nodA*-*lacZ* transcriptional fusion of *R. leguminosarum* bv. *phaseoli* RBL 1283 was also induced by maize kernel exudates (Hungria et al., 1997). A recent proteomic analysis by Vora et al. (2021) showed the expression of NodU protein from *Ensifer* (*Sinorhizobium*) *fredii* NGR234 in response to exudates from maize grown in monoculture and in association with *Cajanus cajan*. The mechanism by which maize activates the expression of some *nod* genes is still unknown, but it would be interesting to explore whether, as by legumes, rhizobial gene induction by maize differs among cultivars. Fungal diversity and microbial interactions with maize depend on the cultivar (Matus-Acuña et al., 2021).

In milpa conditions, there was a dominant effect of bean exudates maybe because they contain flavonoids that are specific to induce *Rhizobium* gene expression (Bolaños-Vásquez and Werner, 1997). Flavonoid biosynthesis genes are regulated by complex mechanisms in plants (Falcone-Ferreyra et al., 2012) and their concentration in exudates may not be the same in bean monoculture and in milpa. We did not quantify them; nevertheless, we found similar expression levels of Ch24-10 *nod* genes in exudates from monoculture beans and milpa. Extracts from soils where corn and beans were grown together are efficient inducers of nodulation genes in *Rhizobium* (Hungria et al., 1997).

We observed similar nodulation in beans grown alone and in association with maize (Figure 5). Contrasting results have been found in the field, where the number and biomass of bean nodules intercropped with maize tends to be higher than monocultured beans (Cardoso et al., 2007; Latati et al., 2013). Nitrogen concentrations in the rhizosphere of bean–maize intercropping system are higher as well; however, this effect varies depending on the physicochemical characteristics of the soil and landraces cultivated (Latati et al., 2013). In the long term, nitrogen supplied by the bean nodules can be beneficial to maize, which needs this element in high quantities for its growth. This metabolic complementarity between rhizobia, bean, and maize could contribute to the high grain yield observed in the field (Cardoso et al., 2007; Zhang et al., 2014; Aguilar-Jiménez et al., 2019).

**FIGURE 8:**
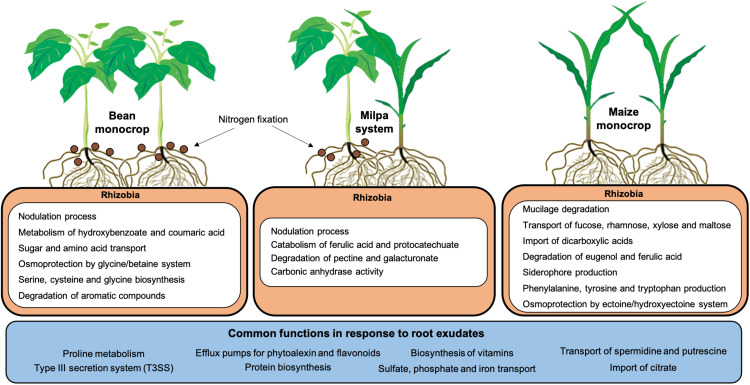
Summary of rhizobial functions associated with monocrops and milpa system. Within the rhizobial cells, upregulated functions induced by each root exudate are shown, while the blue box includes functions that are common in all conditions. The diagram considers the results obtained from transcriptome analysis, evaluation of transcriptional fusions, and enzymatic and nodulation assays.

Some rhizobial genes expressed in maize and bean independent exudates were not the same as those expressed in exudates of maize and bean growing together. Could plant exudate production be affected by exudates from a neighboring plant? Exuded molecules from heterologous or even homologous roots could regulate exudation (Wang et al., 2021). This is an interesting possibility that could be explored. We suggest here that exudates from maize and bean together could have provided a larger diversity of nutrients to *Rhizobium* than single plant exudation assays. However, Ch24-10 may not need to express genes for using nutrients that could be minor components in milpa, but in monocultures, the activation of these genes would give an advantage for rhizobia (Figure 8). In this sense, we could explain why the genes for some sugar transporters and dipeptides were not induced in the laboratory milpa exudate. Minor components that could be left for successive trophic events may not be revealed at 2 h. Rhizobia may participate in the degradation of plant polymers that require more steps for degradation and that would guarantee greater growth by later exploiting the resources derived from degrading pectin, mucilage, or phenolic acids. In agricultural fields, it could be highly advantageous to stimulate bacterial growth in growing plantlets profiting from the rhizobial ability to metabolize nutrients secreted by roots, including those derived from plant walls or border cells.

## Data Availability Statement

The transcriptomic data of this study as BAM files have been deposited in an NCBI BioProject under accession number PRJNA578720 (https://www.ncbi.nlm.nih.gov/bioproject/PRJNA578720).

## Author Contributions

JLA-N and EM-R conceived and designed the experiments. JLA-N performed plant experiments, enzymatic assays, RNA extraction, and bioinformatic and statistical analyses. MR generated bacterial mutants and evaluated their phenotypes in response to root exudates. MGS-M did the protein extraction and carbonic anhydrase activity assays. JLA-N and EM-R analyzed the data and wrote the manuscript. All authors reviewed the article and approved the submitted version.

## Conflict of Interest

The authors declare that the research was conducted in the absence of any commercial or financial relationships that could be construed as a potential conflict of interest.

## Publisher’s Note

All claims expressed in this article are solely those of the authors and do not necessarily represent those of their affiliated organizations, or those of the publisher, the editors and the reviewers. Any product that may be evaluated in this article, or claim that may be made by its manufacturer, is not guaranteed or endorsed by the publisher.

## Footnotes

1
http://eggnog-mapper.embl.de
