# Supplementary material for: Transcriptomic Responses of Rhizobium phaseoli to Root Exudates Reflect Its Capacity to Colonize Maize and Common Bean in an Intercropping System
Source: Front Microbiol. 2021 Oct 28;12:740818. doi: 10.3389/fmicb.2021.740818 (PMC8581550; doi:10.3389/fmicb.2021.740818)
Supplement: Supplementary file 1 [file Data_Sheet_1.pdf]

Supplementary figures

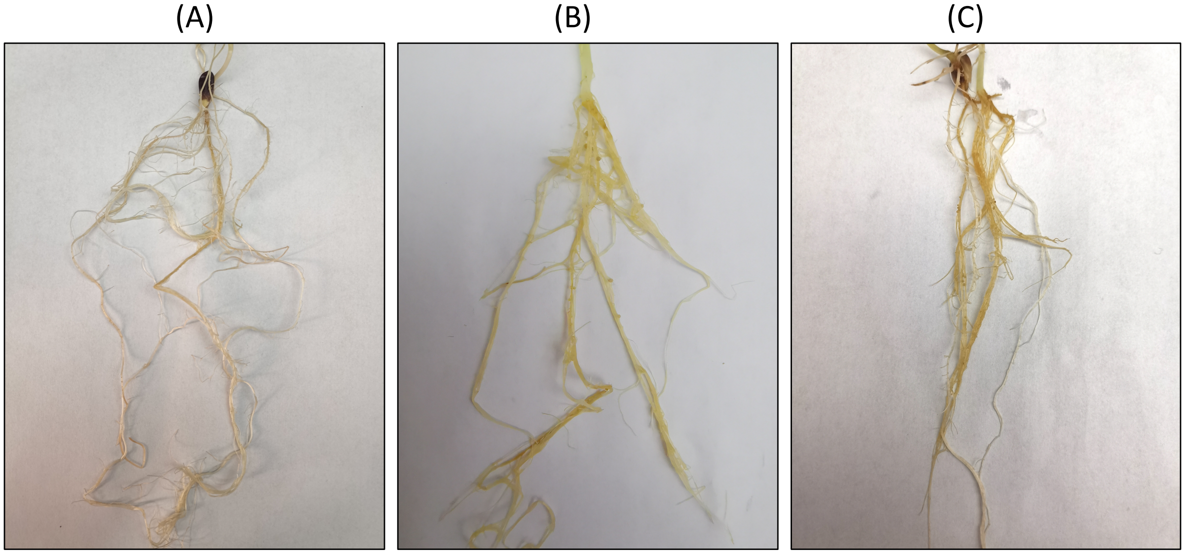

Supplementary Figure 1. Negative controls of β-glucuronidase assays within the roots. Colorless roots of maize (A), bean (B) and milpa(C).

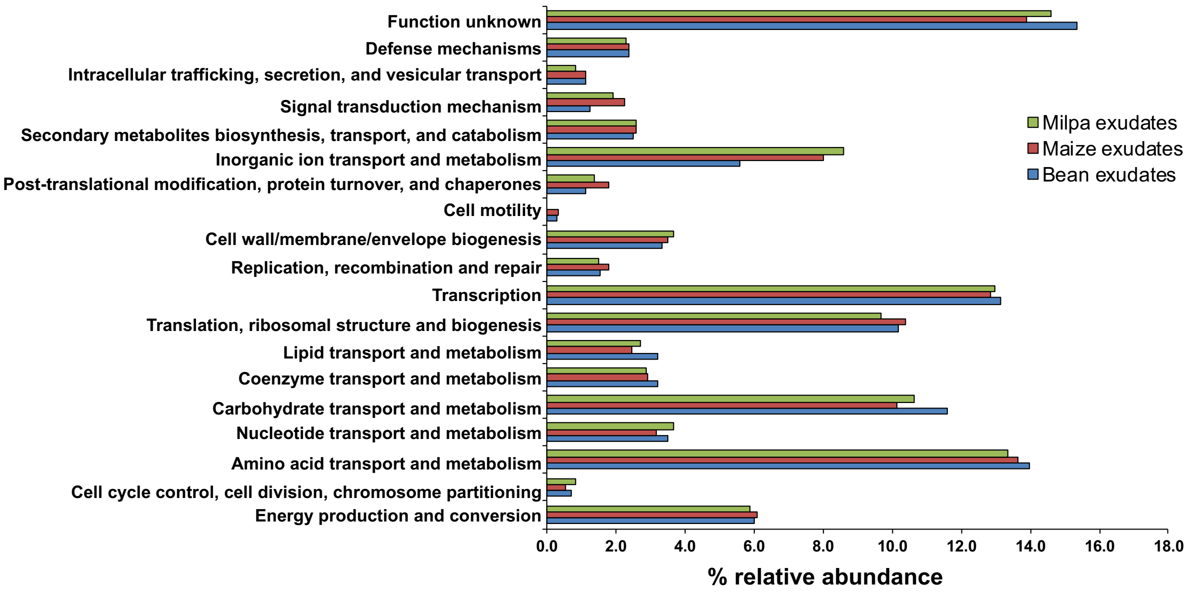

Supplementary Figure 2. Distribution of COG functional categories of overexpressed genes of Ch24-10 in root exudates. The COG classification was carried out in eggNOG-mapper v2 using 694, 923 and 785 up-regulated genes from transcriptomic data of bean, maize and milpa exudates, respectively.

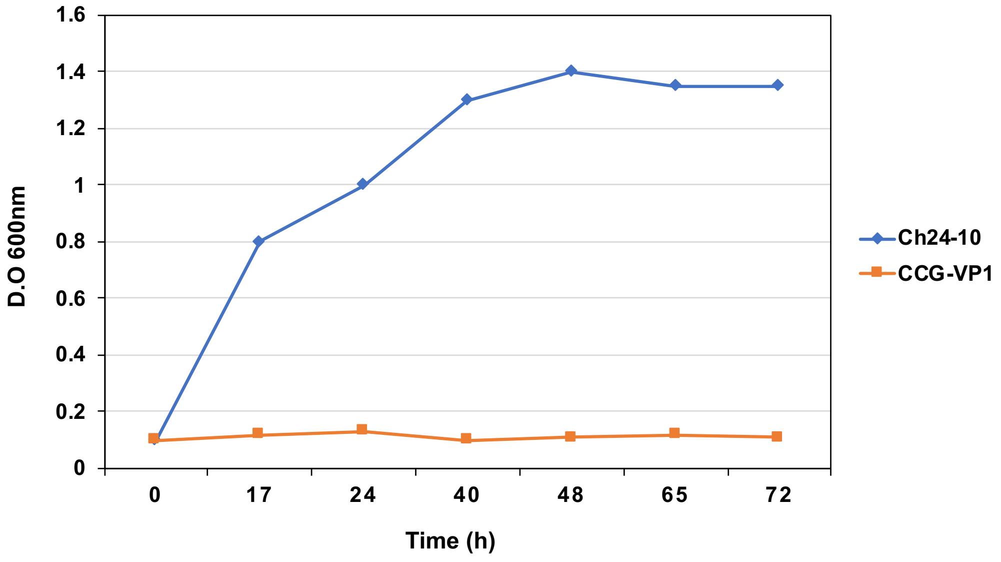

Supplementary Figure 3. Growth curve of the mutant CCG-VP1 (*putA*::*lacZ*) and Ch24-10 (wild type) in minimal medium with L-proline as sole carbon and nitrogen sources.

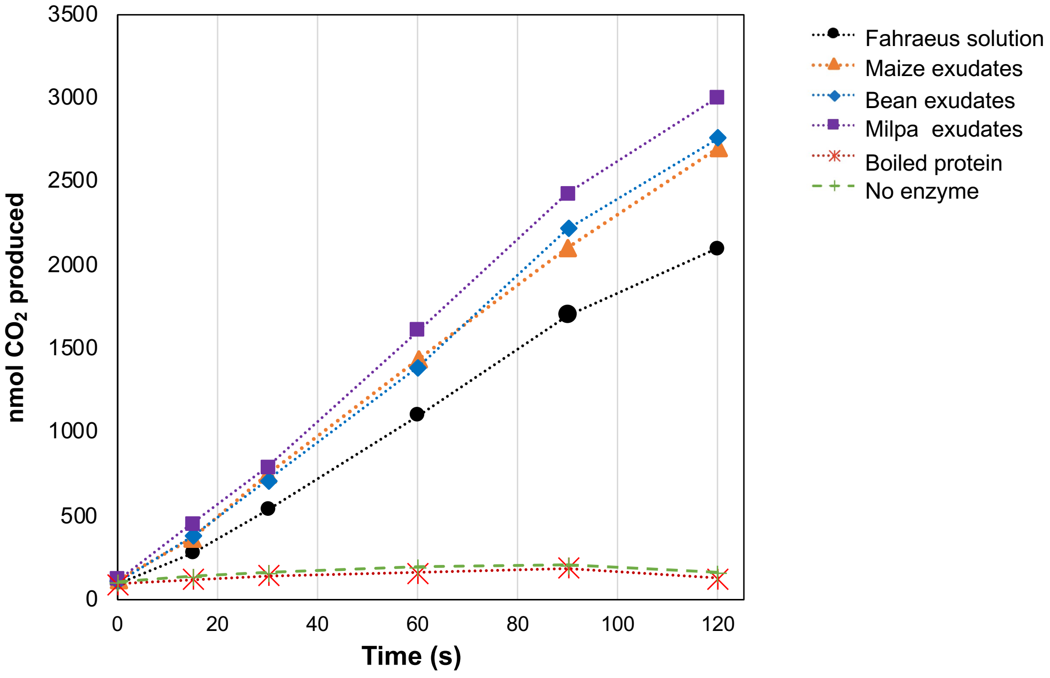

Supplementary Figure 4. Representative data of carbonic anhydrase activity in protein extract from Ch24-10 (after 2 h of incubation with root exudates).

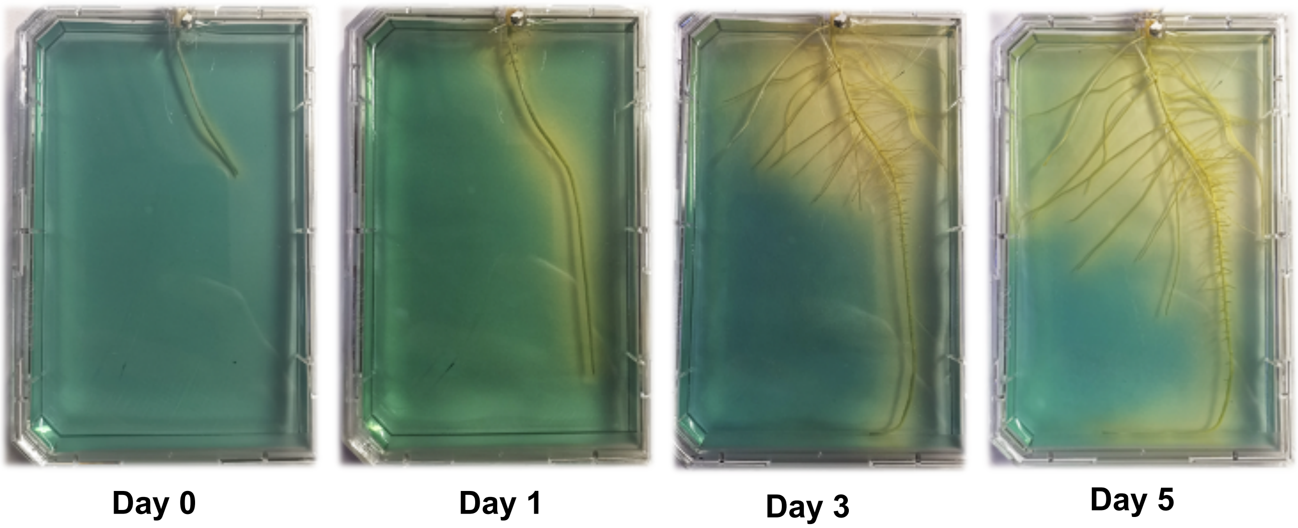

Supplementary Figure 5. Acidification of maize rhizosphere (yellow zone) in Fahraeus medium observed with bromothymol blue. The roots of germinated seeds were introduced into Fahraeus plates (pH 8, agar 0.7%) and the color change was observed every 24 h.

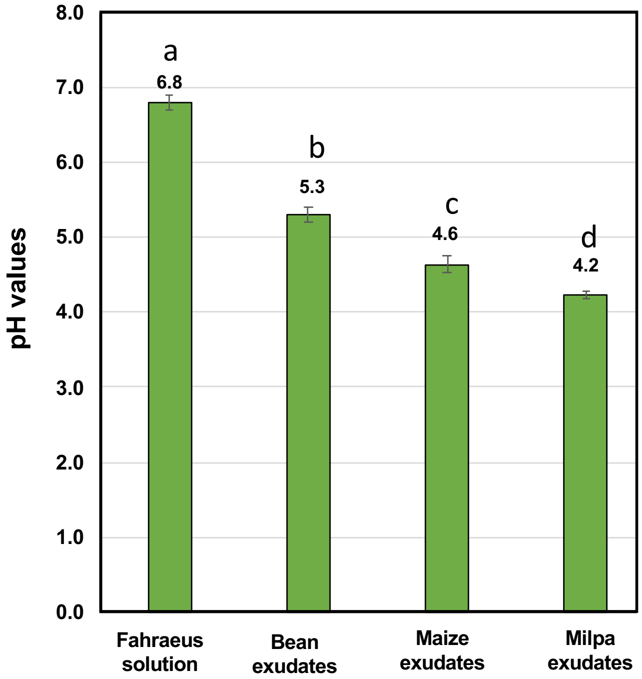

Supplementary Figure 6. pH of exudates from bean, maize and milpa. Exudates from 7-days old plants were collected and the pH was measured with a digital pH meter. Different lower-case letters between treatments show statistically significant differences (p *value* $\leq$ 0.05) according to ANOVA followed by a Tukey's honestly significant difference test. Three replicates for each treatment were performed.

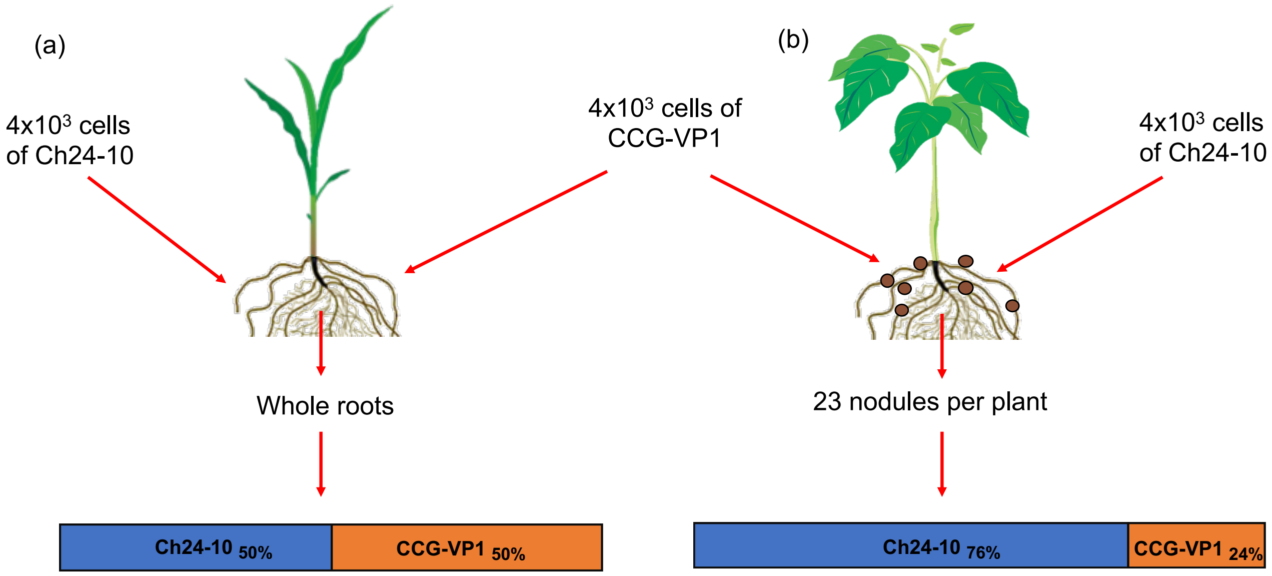

Supplementary Figure 7. Competition assays between strains Ch24-10 and CCG-VP1 to colonize roots. Occupancy rate in (a) maize roots, and (b) bean nodules.
